# Gut microbial clues to bipolar disorder: State‐of‐the‐art review of current findings and future directions

**DOI:** 10.1002/ctm2.146

**Published:** 2020-08-12

**Authors:** Jianbo Lai, Jiajun Jiang, Peifen Zhang, Caixi Xi, Lingling Wu, Xingle Gao, Danhua Zhang, Yanli Du, Qunxiao Li, Xiangyuan Diao, Shaojia Lu, Zheng Wang, Xueqin Song, Shaohua Hu

**Affiliations:** ^1^ Department of Psychiatry the First Affiliated Hospital Zhejiang University School of Medicine Hangzhou China; ^2^ The Key Laboratory of Mental Disorder's Management in Zhejiang Province Hangzhou China; ^3^ Brain Research Institute Zhejiang University Hangzhou China; ^4^ Zhejiang Engineering Center for Mathematical Mental Health Hangzhou China; ^5^ Department of Psychiatry Hangzhou Fuyang Third People's Hospital Hangzhou China; ^6^ Department of Psychiatry the First Hospital of Jiaxing Jiaxing China; ^7^ Department of Psychiatry First Affiliated Hospital of Zhengzhou University Zhengzhou China

**Keywords:** gut microbiome, bipolar disorder, microbiome‐gut‐brain axis

## Abstract

Trillions of microorganisms inhabiting in the human gut play an essential role in maintaining physical and mental health. The connections between gut microbiome and neuropsychiatric diseases have been recently identified. The pathogenesis of bipolar disorder, a spectrum of diseases manifesting with mood and energy fluctuations, also seems to be involved in the bidirectional modulation of the microbiome‐gut‐brain (MGB) axis. In this review, we briefly introduce the concept of MGB axis, and then focus on the previous findings in human studies associated with bipolar disorder. These studies provided preliminary evidences on the gut microbial alterations in bipolar disorder. Limitations in these studies and future directions in this research field, such as fecal microbiome transplantation and microbiome‐targeted therapy, were discussed. A research framework linking gut microbiome to determinants and health‐related outcomes in BD was also proposed. Better characterizing and understanding of gut microbial biosignatures in bipolar patients contribute to clarify the etiology of this intractable disease and pave the new way for treatment innovation.

## Graphical abstract

Previous studies have provided preliminary evidence that gut microbiome is closely linked to mental health in patients with bipolar disorder. Microbiome‐wide association studies help to illuminate the relationship between gut microorganisms and bipolar disorder. Manipulating the gut microbiome appears to be a promising strategy for treating bipolar disorder in the future.

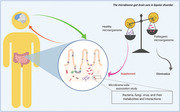

**Abbreviations**
AAP: atypical antipsychotic
BBB: blood‐brain barrier
BD: bipolar disorder
CNS: central nervous system
FMT: fecal microbiome transplantation
GI: gastrointestinal
GWAS: genome‐wide association study
HCs: healthy controls
HPA axis: hypothalamic‐pituitary‐adrenal axis
IBD: inflammatory bowel disease
IBS: irritable bowel syndrome
KYN: kynurenine
LPS: lipopolysaccharide
MAMPs: microbial‐associated molecular patterns
MDD: major depressive disorder
MGB: microbiome‐gut‐brain
NIRS: near‐infrared spectroscopy
NOS: not otherwise specified
OTU: operational taxonomic unit
qPCR: quantitative polymerase chain reaction
SCFA: short‐chain fatty acid
TLR: Toll‐like receptor

## 1 INTRODUCTION

Bipolar disorder (BD) is a common, and severe affective mental illness manifesting with recurrent depressive or manic/hypomanic episodes and affecting approximately 2‐3% of the world's population.^1^ It causes heavy disease burden and leads to significant impairments in cognitive and social functions. In clinical practice, however, the diagnosis of BD is still challenging and lacking specific or objective biomarkers. Delayed or missed diagnosis of BD negatively influences the treatment and prognosis of this disease. Therefore, further clarifying the pathogenesis of BD and identifying biomarkers with potentially diagnostic or prognostic efficacy is of great urgency.

At present, gene‐by‐environment interactions, linking genome, environmental factors, and epigenetic components are considered to be the major cause of BD.^2^ In genetically susceptible individuals, the cumulative effects of environmental factors, such as maternal viral infections during pregnancy, stressful events, and even childhood trauma, would impact the brain regions related to emotion regulation (*eg*, prefrontal cortex and amygdala) by triggering neuroinflammation, causing hyper‐activation of the hypothalamic‐pituitary‐adrenal (HPA) axis and disturbance of neurotransmitter release and kynurenine (KYN) pathway, and eventually contributes to the onset and development of BD.^3^, ^4^, ^5^ In recent years, the human gut ecosystem is also considered to be an essential environmental factor related to neuropsychiatric illnesses, such as major depressive disorder, schizophrenia, and myasthenia gravis.^6^, ^7^, ^8^ Gnotobiotic mice transplanted with feces from depressive or schizophrenic patients correspondingly displayed depression‐like or schizophrenia‐like behaviors.^6^, ^7^ Although supportive evidences from fecal microbiota transplantation (FMT) are still absent for BD, these findings provide primary evidence that gut microbiome could be involved in the pathogenesis of major mental disorders.

Interestingly, antibiomania is a rare clinical phenomenon that refers to antibiotic‐induced hypomania or mania.^9^ Over 10 different antibiotic agents have been reported to be implicated; quinolones and macrolides are the most common suspicious drugs.^10^ A possible explanation for the occurrence of antibiomania is the disruption of the microbiota‐gut‐brain (MGB) axis resulting in inflammation and alterations of cognition, emotion, and behavior.^10^ Moreover, antibiotics use may link to the hospitalization of patients with serious mental illnesses in manic conditions.^11^

The aim of the current review is to briefly introduce the MGB axis, and then focus on previous human studies linking gut microbial clues to BD. We further discuss limitations in these studies and propose future directions in this field of study.

## 2 THE MGB AXIS

The association between gastrointestinal disorders and mental health has been well characterized.^12^ In young and middle‐aged adults, subacute and chronic gastrointestinal (GI) symptoms were reported increasingly in individuals with anxiety, unipolar depression, and BD.^13^ In patients with irritable bowel syndrome (IBS), one of the most common functional GI disorder, reported a greater risk of subsequent BD than those without IBS.^14^, ^15^ Furthermore, inflammatory bowel disease (IBD), mainly consisting of Crohn's disease and ulcerative colitis, is also associated with higher risk of comorbid BD condition.^16^ Various studies have revealed IBD was linked to disturbed gut microbial compositions and functions.^17^, ^18^ These findings indicated an overlapping biological pathway shared by BD and GI pathologies.

The human gut microbiome is dynamic and complex, harboring a huge amount of microorganisms, including bacteria, fungi, bacteriophages, and other virus.^19^, ^20^ These microorganisms can produce various biochemical molecules, such as hormones, cytokines, and neurotransmitters, which interact with the central nervous system (CNS) through endocrine system, neuroimmune network, vagus nerve, or enteric nervous pathways.^19^, ^21^ Take the vagus nerve as an example, it is an important bridge between the CNS and the enteric nerve system. Vagal nerve stimulation has been proved to be an effective and safe therapy for treatment‐resistant depression.^22^ Vagotomy can alleviate the brain activation following oral supplement of psychoactive bacteria, such as *Lactobacillus rhamnosus* and *Bifidobacterium longum*, indicating the engagement of vagal‐dependent pathway in the MGB axis regulation.^23^, ^24^

This MGB axis is bidirectional and essential to maintain systemic homeostasis. On the one hand, microbiome‐derived bacterial fermentation products (*eg*, short‐chain fatty acids) released into systemic circulation can be transmitted through the blood‐brain barrier (BBB) and influence the brain function directly, such as controlling microglial maturity.^25^ In patients with severe mental illnesses, the integrity of gastrointestinal (GI) barrier and BBB may be impaired and their permeability was thus increased.^26^, ^27^ This facilitates the transmission of bacterial metabolites from intestinal lumen and portal circulation into the CNS, eventually resulting in systemic and central physiological dysfunction.^21^ On the other hand, the CNS can affect the gut microbes by releasing signals for food selection and food intake, regulating autonomic nervous system and controlling hypothalamic‐pituitary‐adrenal endocrine axis.^19^, ^28^

Notably, the emergence of immunoneuropsychiatry witnesses the essential role of immune processes in maintaining CNS homeostasis and resilience.^29^ Previous studies have suggested a chronic, persistent, and low‐level inflammation underlying the pathogenesis of mood disorders.^29^, ^30^ Of note, the gut ecosystem could be a major reservoir for this inflammatory process.^31^ Take lipopolysaccharide (LPS), for example, as a common component of microbial‐associated molecular patterns, transferring LPS into the systemic circulation can be recognized by TLRs on immune cells, which trigger cellular inflammatory reactions and release cytokines, chemokines, interferons, and other immune mediators.^32^, ^33^ Entrance of inflammatory factors into the CNS may disrupt immunological balance and cause emotional, behavioral, and cognitive symptoms in neuropsychiatric diseases.^32^, ^34^

Additionally, neuroinflammation stimulates the expression of KYN enzymes and modulates the metabolic degradation of tryptophan in the CNS.^35^ Tryptophan is an essential amino acid that in vivo can be converted into various substances in an enzyme‐dependent manner, including neurotransmitters (eg, serotonin and melatonin) and KYN metabolites (eg, kynurenic acid, quinolinic acid, and xanthurenic acid).^36^, ^37^ Generally, kynurenic acid is considered to be neuroprotective while quinolinic acid has neurotoxic effects. Proinflammatory mediators, such as IL‐6, IL‐1β, and TNF‐α, on the one hand, are able to upregulate the serotonin transporter (SERT) function and diminish serotonin pathway; on the other hand, IFN‐γ induces the activation of indoleamine 2,3‐dioxygenase (IDO) and favors the generation of KYN metabolites, especially toward the neurotoxic branch.^36^, ^37^

**FIGURE 1:**
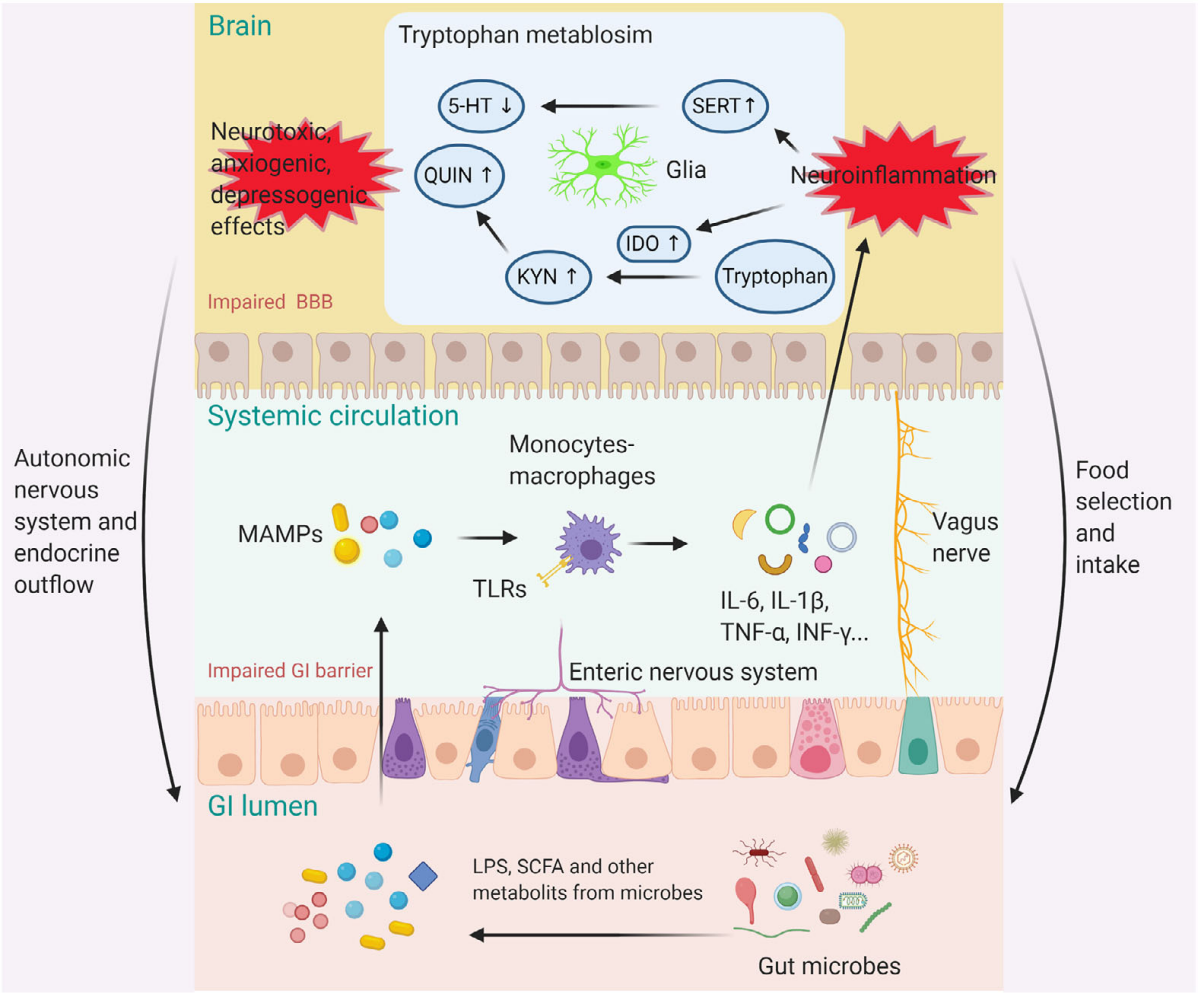
Graphic paradigm for the microbiome‐gut‐brain axis regulation in mood disorders. The bidirectional modulation between the gut microbiome and the CNS depends dominantly on the neuroimmune, neuroendocrine and nervous systems. In patients with mood disorders, the GI and blood‐brain barriers are possibly compromised, facilitating MAMPs to enter the systemic circulation and the CNS. MAMPs recognized by receptors (*eg*, TLRs) on peripheral immune cells, such as monocytes‐macrophages, cause peripheral and inflammatory reactions. Peripheral inflammatory mediators, such as IL‐6, IL‐1β, TNF‐α, and INF‐γ, penetrate the compromised BBB and elicit dysregulation of central immune process in mood‐regulating areas (*eg*, hippocampus and prefrontal cortex). Neuroinflammation further modulates the tryptophan metabolism in the brain, by enhancing the activity of serotonin transporter and IDO enzyme, causing reduced level of serotonin in the synaptic cleft and elevated level of the neurotoxic component, quinolinic acid. The neuroinflammation and its influence on tryptophan metabolism, may contribute to the emotion, behavior and cognitive abnormalities in mood disorders. Abbreviations: LPS, lipopolysaccharide; TLR, toll‐like receptor; MAMPs, microbial‐associated molecular patterns; CNS, central nervous system; SCFA, short‐chain fatty acid; GI, gastrointestinal; BBB, blood‐brain barrier; SERT, serotonin transporter; IDO, indoleamine 2,3‐dioxygenase; KYN, kynurenine; QUIN, quinolinic acid; 5‐HT, serotonin

Collectively, clarifying how the MGB axis works in BD, especially microbiome‐immune‐kynurenine interactions for regulating brain function and behaviors, paves the new way to understand the pathogenesis of BD and promotes the development of novel treatment strategies targeting at this thoroughfare (Figure 1).

## 3 GUT MICROBIOME AND BD IN HUMAN STUDIES

Up to the present, there are 12 clinical studies investigating the gut microbiome in BD individuals (Table 1). We collected and summarized data from these studies, including sample size, age, sex ratio, disease status, subtypes, medication history, gut microbial diversity, and composition, and the region or country where the study was conducted. These studies provide preliminary evidences on the gut microbial alterations in BD patients.

**TABLE 1:** Researches characterizing the gut microbiota in human through qPCR, 16s rRNA, or shotgun metagenomic sequencing

Number	Sample size	Mean age	Female N (%)	Disease phases or severity	Type of BD	Medications	Alpha diversity	Taxonomic difference	Publication year/Country (References)
**1**	Patient: 23	45.4	16 (69.6%)	Not controlled	BD I: 15 BD II: 8	Not controlled	BD patients had reduced OTU abundance than HC	(1) A *Clostridiaceae* OTU ↑ (2) Elevated *Collinsella* in BD II relative to BD I	2019 Canada (44)
	HC: 23	43.75	16 (69.6%)						
**2**	Patient:115	50.2	83 (72.2%)	Not controlled	BD I: 76 BD II: 29 NOS: 10	Not controlled	Not evaluated	(1) *Faecalibacterium* ↓	2017 America (39)
	HC: 64	48.6	40 (62.5%)					(2) *Ruminococcaceae* family ↓	
**3**	Patient: 32	41.3	14 (43.8%)	13 patients in a depressive episode	BD I: all	Not controlled	No significant difference between BD and HC	(1) Phylum *Actinobacteria*, Class *Coriobacteria*, Order *Coriobacteriales*, Family *Coriobacteriaceae* ↑ (2) Family *Ruminococcaceae*, Genus *Faecalibacterium* ↓	2018 Australia (45)
	HC: 10	31.4	6 (62.5%)						
**4**	Patient: 39	40.3	22 (56.4%)	Not controlled	BD I: 13 BD II: 26	Not controlled	Not evaluated	No significant difference in *Bifidobacterium* or *Lactobacillus* counts between 2 groups	2019 Japan (40)
	HC: 58	43.1	36 (62.1%)						
**5**	28 patents with BD; 9 patients with schizophrenia or schizoaffective disorder^a^	Not controlled	BD I: 19 BD II: 9	AAP‐treated group or mood stabilizer‐treated group	Decreased diversity in AAP‐treated females than non‐treated females	(1) Fractional representation of *Alistipes* in non‐AAP users ↑	2019 America (46)		
						(2) Phylum *Actinobacteria* in AAP‐treated patients with resistant starch administration ↑			
**6**	Patient: 32	41.7	7 (21.9%)	13 in depression;	unknown	Not controlled	Lower alpha‐diversity in patients with current depression	None	2019 Australia (51)
	HC: 0	/	/	19 in euthymia					
**7**	Patient: 113	31.0	70 (62.5%)	Not controlled	BD I: 44 BD II: 65	Not controlled	No difference in bacterial diversity between BD and HC	(1) Presence of *Flavonifractor* in patients having BD ↑	2019 Denmark (49)
	HC: 77	29.0	47 (61%)						
**8**	AAP‐treated: 69	51.7	48 (69.6%)	Not controlled	unknown	Not controlled	AAP‐treated females showed lower species diversity compared to non‐treated females	(1) *Lachnospiraceae* in AAP‐treated patients ↑ (2) *Akkermansia* and *Sutterella* in non‐treated patients ↑	2017 America (47)
	Non‐treated: 46	46.0	34 (73.9%)						
**9**	Patient: 36	32.6	15 (41.7%)	All patients in depressive episodes	BD I: 10	Quetiapine monotherapy for one month	Not evaluated	(1) *Faecalibacterium prausnitzii*, *Bacteroides–Prevotella*, *Atopobium Cluster*, *Enterobacter spp* and *Clostridium Cluster IV* in untreated patients ↑	2019 China (38)
	HC: 27	28.9	12 (44.4%)		BD II: 26				
**10**	Patient: 52	24.2	25 (48.1%)	All patients in depressive episodes	BD I: 12 BD II: 38 NOS: 2	Quetiapine monotherapy for one month	Lower α‐diversity in untreated patients; quetiapine therapy did not change α‐diversity	(1) Altered bacterial composition in untreated patients (2) Quetiapine treatment changed the bacterial composition (3) Gut microbial biomarkers may help to diagnose BD and predict treatment outcome	2019 China (48)
	HC: 45	36.3	22 (48.9%)						
**11**	Patient: 30	38.4	15 (50.0%)	All patients in depressive episodes	unknown	Not controlled	No difference in bacterial α‐diversity between BD and HC	(1) *Phyla Firmicutes*, *Actinobacteria* and *Proteobacteria* ↑; *Bacteroidetes*↓in the BD group (2) At genus level, abundance of various bacteria was changed	2019 China (42)
	HC: 30	39.5	16 (53.3%)						
**12**	Patient: 169	25.6	84 (49.7%)	All patients in depressive episodes	unknown	Not controlled	Indices of Ace and Chao were decreased in BD relative to HCs	(1) Phyla *Proteobacteria*↑, *Bacteroidetes*↓, and Family *Pseudomonadaceae* ↑in the BD group (2) BD shows disturbed covarying OTUs belonging to *Lachnospiraceae*, *Prevotellaceae*, and *Ruminococcaceae* families.	2020 China (43)
	HC: 171	26.9	100 (58.5%)						

a No healthy controls were included in this study. All patients were medicated and classified into the AAP‐treated group and non‐AAP group. The samples in this study were divided into the discovery group and the validation group. The data presented in this table is based on the discovery group. Abbreviations: OTU, operational taxonomic unit; BD, bipolar disorder; HC, healthy control; AAP, atypical antipsychotic; NOS, not otherwise specified; qPCR, quantitative transcription‐polymerase chain reaction.

### 3.1 Changes of gut microbial compositions in BD

Our research group previously collected fecal samples from 36 BD subjects and 27 HCs and examined the abundance of 10 common bacterial species with quantitative polymerase chain reaction (qPCR). Compared to HCs, *Faecalibacterium prausnitzii*, *Bacteroides–Prevotella*, *Atopobium Cluster*, *Enterobacter spp*, and *Clostridium Cluster IV* counts were increased in BD subjects, while log_10_(B/E), the ratio of *Bifidobacteria* to *Enterobacteriaceae*, was decreased. B/E value represented the microbial colonization resistance of the bowel. In this study, notably, we applied near‐infrared spectroscopy (NIRS) to assess brain function. A new concept of brain‐gut coefficient of balance (B‐G_CB_), the ratio of oxygenated hemoglobin to B/E, was proposed. We found log_10_(B‐G_CB_) was positively correlated with peripheral CD3^+^ T‐cell proportion.^38^ These findings indicated the intrinsic balance of the MGB axis might be regulated by the immune pathway. Using 16S rRNA sequencing, Evans et al analyzed stool microbiome from 115 BD patients and 64 HCs. They found that the fractional representation of *Faecalibacterium* was decreased and associated with better self‐reported burden of disease measured by regard to sleep, depression, anxiety, and mania.^39^ Aizawa et al examined *Bifidobacterium* and *Lactobacillus* counts in fecal samples from 39 BD patients and 58 HCs. A negative correlation between *Lactobacillus* counts and sleep, as well as *Bifidobacterium* counts and cortisol levels, was reported. However, no significant difference of abundance was identified in either bacterium between the two groups.^40^ In addition, researchers extended the classic gene set enrichment analysis to the published data set of genome‐wide association study (GWAS) of gut microbiome and revealed Genus *Desulfovibrio* was likely to be associated with BD.^41^ These studies preliminarily showed an altered gut microbial compositions in depressed BD patients. However, due to the clinical heterogeneity and methodological differences, the findings were inconsistent across different studies.

### 3.2 Comparisons of gut microbiota in BD and MDD

Rong et al first performed the shotgun metagenomic sequencing to compare the differences of gut microbiome in currently depressed individuals with BD and major depressive disorder (MDD). Compared to HCs, decreased microbial alpha‐diversity was observed in MDD patients, but not in BD patients. In addition, abundances of phyla *Firmicutes* and *Actinobacteria* were multiplied but *Bacteroidetes* was reduced in both MDD and BD groups. However, phyla *Proteobacteria* was increased only in BD patients, not in MDD patients.^42^ In a recently published study, we compared microbial compositions from 165 subjects with MDD, 217 with BD and 217 HCs by 16S rRNA sequencing. Decreased species richness was found in BD patients relative to HCs, but not in MDD patients. Relative abundance of microbial compositions was different at the phylum and family levels between MDD and BD patients. Moreover, a microbial operational taxonomic unit (OTU) panel was identified that could efficiently distinguish between bipolar and unipolar depression.^43^ These two studies provided preliminary evidence on biological disparities between MDD and BD, as reflected by a different microbiome in the human intestine.

### 3.3 Associations of gut microbiome with clinical/laboratory characteristics in BD

The relationship between gut microbiome and clinical or laboratory profiles has also been explored. McIntyre et al compared the gut microbiome profiles in 23 BD patients to 23 healthy controls (HCs). Decreased microbial diversity and a greater abundance of a *Clostridiaceae* OTU was observed in BD patients. In addition, a greater abundance of in *Collinsella* was reported in BD‐II relative to BD‐I subjects, indicating a potential divergence of microbial composition in different subtypes of BD.^44^ Painold et al performed 16s rRNA sequencing of stool samples from 32 BD patients and 10 HCs. This study found microbial alpha‐diversity was negatively correlated with illness duration. Linear discriminant analysis effect size (LEfSe) revealed the phylum *Actinobacteria* and the class *Coriobacteria* were more abundant in BD subjects, while *Ruminococcaceae* and *Faecalibacterium* were more abundant in HCs. As determined by Beck's Depression Inventory cut‐off score of 18, the family of *Enterobacteriaceae* was more abundant in patients with clinically relevant depressive symptoms, while the family of *Clostridiaceae* and the genus *Roseburia* were more abundant in recovered BD patients. Specific bacterial clades were associated with inflammatory status, serum lipids, tryptophan level, oxidative stress, depressive symptoms, and metabolic syndrome, indicating a complex interactive network between host health and gut microbiome.^45^ These findings suggested that specific bacteria species in the intestine were associated with different BD subtypes and severity of depression, and might affect host immune, metabolic and oxidative stress processes.

### 3.4 Effects of antipsychotics on gut microbiota in BD

The effects of antipsychotics on the gut microbiome have also been investigated. A recent study from Flowers et al recruited 28 patents with BD and nine with schizophrenia or schizoaffective disorder. Compared to AAP nonusers, AAP users reported lower fractional representation of *Alistipes*. Compared to female patients not treated with AAP, the one who was treated with AAP exhibited decreased microbial diversity. Furthermore, resistant starch supplement increased the abundance of the phylum *Actinobacteria* in AAP‐treated patients.^46^ Another study by Flowers et al obtained fecal microbiome from 49 AAP‐treated and 68 non‐AAP treated BD patients. In female patients, AAP treatment was associated with decreased species diversity. *Lachnospiraceae* was more abundant in AAP‐treated patients, while *Akkermansia* and *Sutterella* was more abundant in nontreated patients.^47^ These two studies indicated AAP use could be a determinant on the gut microbial diversity and composition, especially in female patients. In another study, we used 16s rRNA sequencing of fecal microbiome from 52 depressed BD patients and 45 HCs. Compared to HCs, decreased microbial diversity and altered microbial composition were shown in untreated BD patients. Following 1‐month of quetiapine treatment, bacterial α‐diversity did not change significantly, while their composition changed in BD patients. Moreover, random classification models revealed that microbial OTUs can help to classify patients from HCs and even predict treatment outcome of quetiapine. This is the first study to explore the effects of AAP monotherapy (quetiapine) on gut microbial structure and composition.^48^ Future studies are warranted to investigate the long‐term outcomes of certain antipsychotic on gut microbial communities and compare the effects of different antipsychotics.

### 3.5 Twins or relatives study of gut microbiome in BD

Only one study reported that the gut microbial community membership and structure was different among patients with BD, their unaffected first‐degree relatives and HCs. However, unaffected relatives and HCs were not distinguishable in community membership or structure. Presence of *Flavonifractor*, a bacterial genus that may participant in oxidative stress and inflammation, in patients with newly diagnosed BD was associated with smoking and female sex.^49^ The only longitudinal study investigated fecal microbiome in one pair of monozygotic twins discordant for BD and found changes in gut bacterial community structure, composition, and functional profiles during active depressive state, which could be attenuated when the patient achieved full remission.^50^ The gut microbiome could be an endophenotype of BD, but the interactions between host genes and gut microbiome remain almost unexplored.

### 3.6 Gut microbiome and host epigenetic modification in BD

Interestingly, Bengesser et al reported gut microbial diversity and evenness correlated negatively with epigenetic alterations of the molecular clock gene *ARNTL*, methylation on the specific CpG site cg05733463. Furthermore, compared to euthymic BD patients, depressed individuals exhibited lower bacterial α‐diversity.^51^ This is the first and only study indicating a possible link between gut microbiome and epigenetic modification of BD‐related genes. Given the involvement of the MGB axis in the neurodevelopment of BD, the gut microbiome may act as an essential regulator to modify or manipulate the expression of BD risk genes.

Despite these studies being preliminary, their findings demonstrate an essential role of the MGB axis involved in the pathogenesis of BD, indicating the diagnostic or prognostic potentials of microbial markers in clinical practice. Decreased α‐diversity of gut microbiome was reported in most BD cohorts. Individual differences, including gender, age, and diet, and disease features, including subtypes, course, and severity of illness and AAP use, could all be determinant factors of gut microbial ecosystem. Of special note, some studies have implied gender differences in gut microbial diversity and compositions. Future researches are warranted to clarify how this axis arranges to bring about the occurrence and development of this intractable mental disorder.

## 4 LIMITATIONS OF PREVIOUS STUDIES

Although relevant studies are accumulating in recent years, investigations of gut microbiome in BD are still in its infancy. Communicating mechanisms between the brain and gut microbiome in BD individuals have not been explored in details. Most studies focus on the determinant role of gut microbiome in maintaining mental health and its capacity to influence parameters significant to disease severity and pathology, but inevitably ignore that individual behaviors associated with emotion instability, physical discomfort, social interaction, and food intake can also result changes in gut flora. Additionally, there are several limitations in these studies that warrant special cautions to interpret research findings. First, the demographic characteristics of participants were not completely evaluated. In addition to race, age, and gender, other factors, such as body mass index, smoking, exercise, diet, antimicrobials use or other medications with endocrine or immunoregulatory effects should also be recorded. Dietary patterns and food preferences are important determinants on gut microbiome by involving in food digestion, nutrient absorption, shaping the intestinal immune system, and releasing bioactive metabolites.^52^ In BD patients with unstable mood, disordered eating behaviors, with either deceased or increased appetite, are always observed. However, it remains unknown whether changes in eating patterns are associated with specific gut microbial communities in BD patients. Second, the clinical features of disease were not well characterized, including disease phase, age onset, course of illness, symptom severity, psychotic symptoms, suicide risks, and comorbid physical illnesses. For example, most previous studies failed to investigate the potential differences of microbial diversity and compositions between type I BD and type II BD individuals. Given the varying clinical manifestations in patients with BD, it seems necessary to clarify the internal relationship between clinical features of BD and specific microbial clades. Third, most studies were in a naturalistic, cross‐sectional design with a small sample size and not strictly controlled the psychotropic medications. However, previous studies have indicated a possible influence of antipsychotics on gut microbiota.^46^, ^47^, ^48^ Long‐term observations of microbial signatures are needed to understand the causal relationship between gut microbiome and BD. Fourth, a major obstacle of integrating previous findings is the heterogeneity in fecal sample collection, storage, and analysis across different studies.^53^, ^54^ A previous study tested 21 representative DNA extraction protocols with differences due to library preparation and sample storage, and found that DNA extraction had the largest effect on the results of metagenomic analysis.^53^ Therefore, a standardized fecal sample processing pipeline with consensus should be established to guarantee the sample and data quality.^54^ Fifth, but not the last, no studies have ever focused on the characteristics of gut microbiome in young patients with BD (*eg*, children and adolescence), as well as those with refractory BD. Decoding the gut microbiome in these special populations may help to understand the etiology of BD.

## 5 FUTURE DIRECTIONS

The interactions between gut microbiome and host health are dynamic, variable, and multifaceted. Bacteria residing in the intestine participate closely in physiological or pathological processes, such as cytokine release, neurotransmitter production, tryptophan metabolism, and oxidative stress, by activating the hypothalamic‐pituitary‐adrenal (HPA) axis and entero‐endocrine/immune pathways and stimulating the ascending neural pathway via vagus nerve.^55^, ^56^, ^57^ Based on previous findings, it seems credible that the MGB axis is involved in the pathogenesis of BD and the gut microbiome has emerged as a new marker for mental health. However, great efforts are urgently needed to untangle the operating mechanisms of the MGB axis in BD.

**FIGURE 2:**
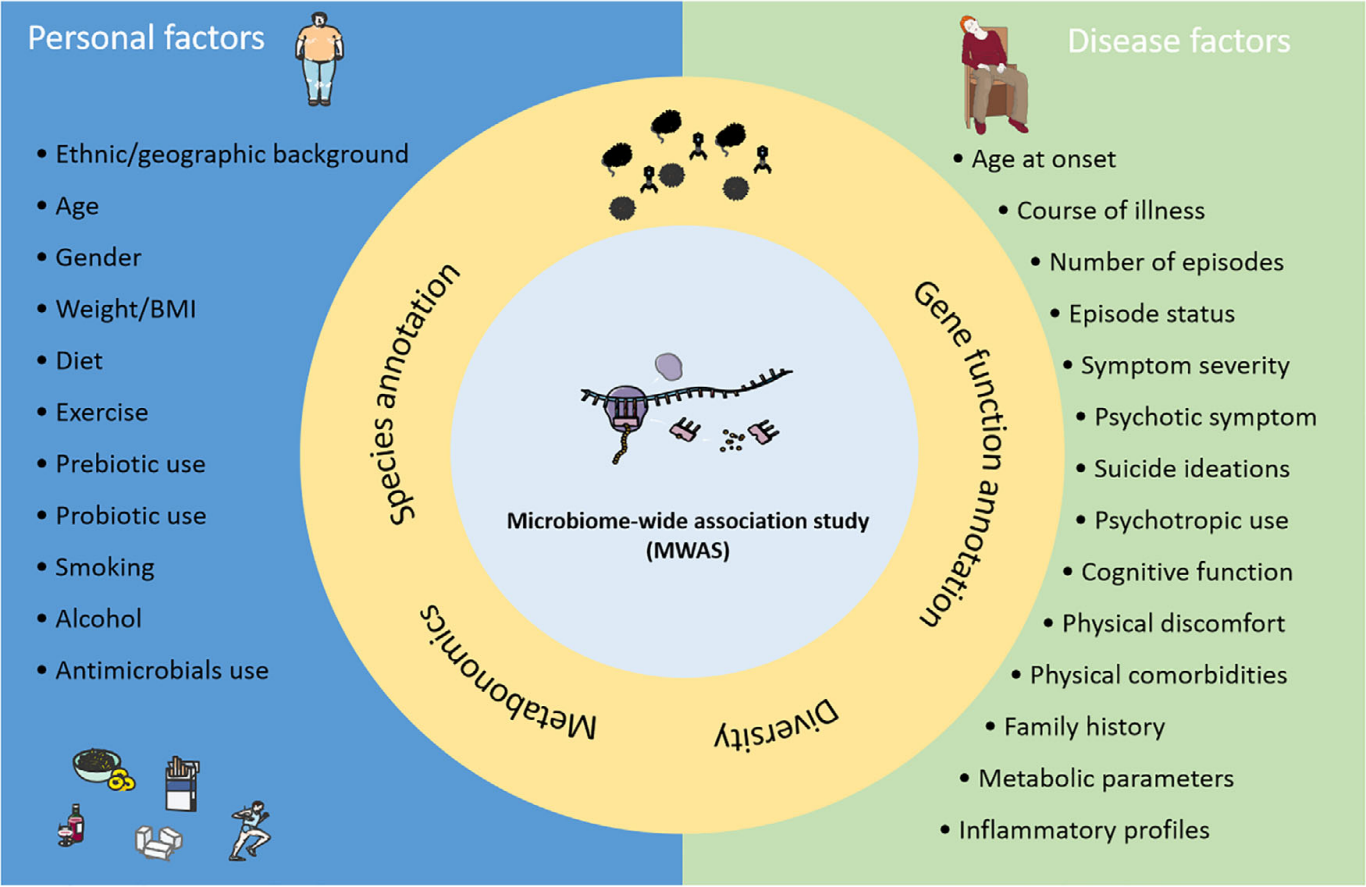
A research framework for investigating the links between determinants of gut microbiome and health‐related outcomes in BD. Standardized sequencing and analysis of gut microbiome in BD patients with either 16s rRNA gene or metagenomic sequencing is necessary to annotate the species and gene functions. The gut microbial diversity, variations and compositions may be driven by environmental and personal factors, and may link to specific clinical features, metabolic and inflammatory measurements in patients with BD. Well characterization of individual and disease factors is important for investigating gut microbiome in BD

**FIGURE 3:**
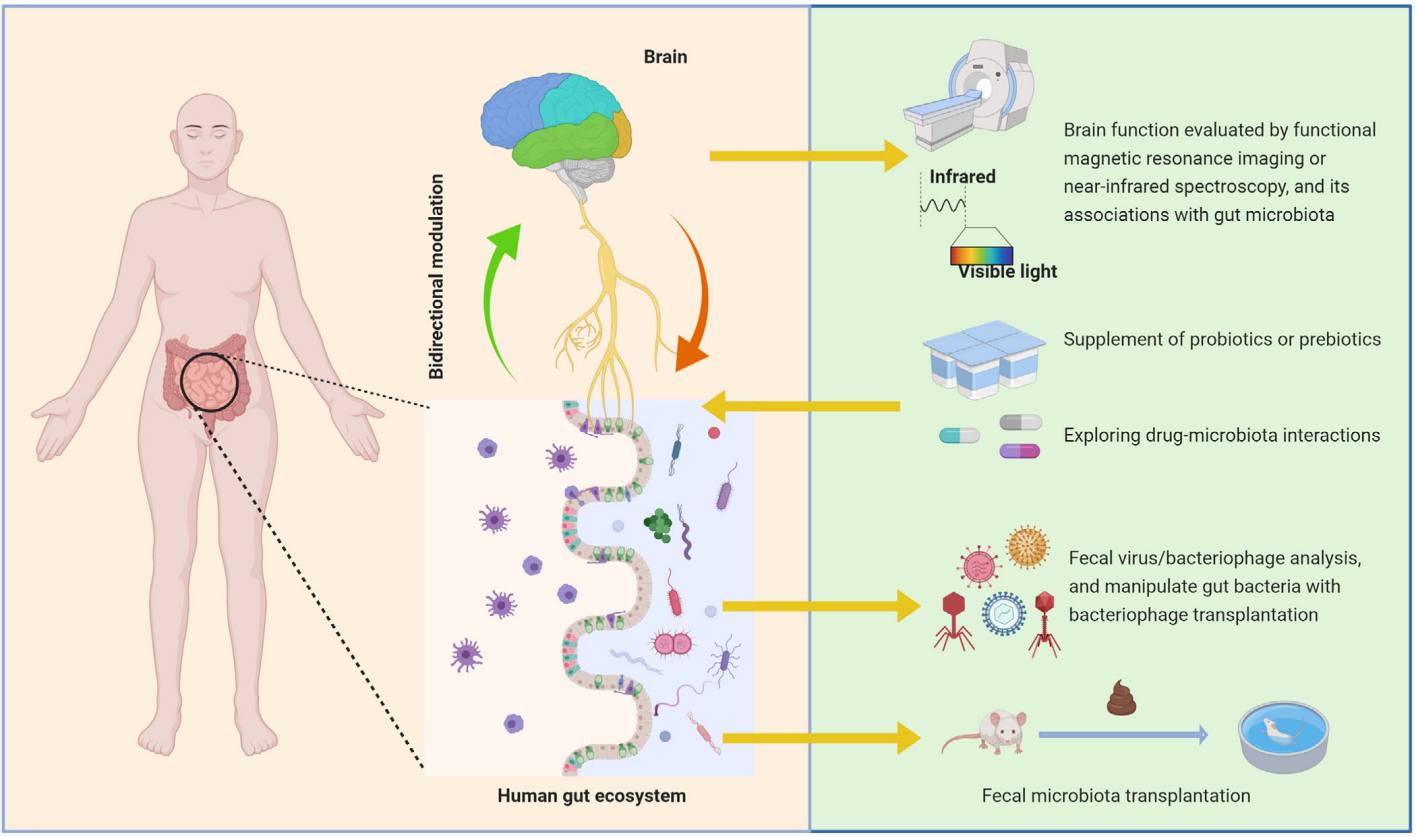
Strategies for future basic and clinical research on gut microbiome in BD individuals. In addition to clarify the bacterial species in the human intestine, the gut viral populations, especially for bacteriophages, should also be investigated. Fecal microbiota transplantation is also needed to verify the role of gut microbiome in mood regulation. Synchronous evaluation of gut microbiome and brain function by functional magnetic resonance imaging or near‐infrared spectroscopy and analysis of their associations help to better understand the microbiota‐gut‐brain regulation. Due to inadequate evidence, the auxiliary effect of prebiotic or probiotic supplement in treating BD patients is worth more investigations. Pharmacomicrobiomics aiming at clarifying the interactions between gut microbiome and pharmacotherapy can facilitate individualized treatment in the future

According to the disease features of BD, we herein propose a research framework to guide the future studies of gut microbiome in BD (Figure 2). Well characterization of personal and disease factors that link to gut microbiome is of great importance to improve comparability of different studies and facilitate meta‐analysis. Recently, the new concept of “microbiome‐wide association study,” an analogue to GWAS, has been proposed.^58^ Obviously, big data, multi‐omic studies that integrate host genomics, microbiome genomics, metabonomics, and brain connectomics will help to depict a complicated communicating network in the MGB axis. The gut microbiome may affect the expression or epigenetic modification of host genes, and thus participate in various physiological or pathological processes linking to human health or diseases. However, in the near future, several research works are necessary to implement in priority to clarify the relationship between gut microbiome and BD, such as FMT in animals, intestine bacteriophage analysis, and microbiome‐targeted therapy (Figure 3).

### 5.1 Fecal microbiota transplantation

FMT has been proven as a safe and effective therapeutic option for *Clostridium difficile* infection.^59^ Recently, promising evidences have also shed light on the potential of FMT in managing other diseases associated with gut microbial alterations, such as IBD, IBS, obesity, multidrug resistant infections, and neuropsychiatric illnesses.^60^ In animal study, transplantation of feces from mentally ill patients have shown to sufficiently elicit disease‐specific behavior phenotypes, such as schizophrenia, MDD, and autism.^6^, ^7^, ^61^, ^62^, ^63^ At present, however, no published data have ever explored the role of feces from BD patients on mice behaviors. Therefore, the first step to be taken in BD research is to validate the efficacy of patient‐derived feces to cause BD‐like symptoms in animal models, not only limiting to depressive behaviors, but also mania‐like manifestations.

### 5.2 Intestine bacteriophage analysis

Virome living in the intestine, especially bacteriophages, is also one of the major microbial populations of the gut microbiome. Advanced sequencing methods have made it possible to figure out the entire viral genomes in the intestine.^64^ A longitudinal metagenomic analysis of fecal virus in healthy adults revealed that the gut bacteriophages were highly diverse, stable, and individual‐specific, and closely affiliated with the gut predominant bacteria taxa.^65^ Gut virome alterations were observed in patients with inflammatory bowel disease.^66^, ^67^ Fecal virome transplantation in a murine model can alleviate metabolic symptoms of type 2 diabetes and obesity.^68^ Given its role in modulating bacterial colonization, bacteriophages can be harnessed to modulate the bacterial species via interbacterial interactions. Therefore, decoding the bacteriophages in BD patients is beneficial for developing future biomarkers and therapeutics.

### 5.3 Microbiome and pharmacotherapy outcome

The interactions between gut microbiome and pharmacotherapy have been currently recognized as an important determinant that influences the treatment outcomes. The concept of “Pharmacomicrobiomics” has become a popular research field, especially in the anticancer study.^69^, ^70^ The anticancer treatments (*eg*, chemotherapy or immunotherapeutic drugs) can affect the gut microbial compositions and diversity. Conversely, the gut microbiome also can modulate the drug efficacy and toxicity.^69^ In previous studies of BD patients, AAP use was found to be associated with altered microbial compositions, and females with AAP use showed decreased bacterial diversity.^46^, ^47^ One of our previous study has also reported the impact of 4‐week quetiapine monotherapy on gut microbial diversity and compositions.^48^ A random forest model constructed on microbial OTUs displayed favorable predictive efficacy on 1‐month quetiapine treatment in depressed BD patients.^48^ In addition to AAPs, other psychotropic agents, such as antidepressants and anticonvulsants, have also exhibited antimicrobial properties. Selective serotonin re‐uptake inhibitors (*eg*, fluoxetine, paroxetine, and sertraline), a widely used class of antidepressant, may manifest with varying antimicrobial effects against Gram‐positive and Gram‐negative bacterial species, which has been reviewed in details in recently published studies.^71^, ^72^ Valproic acid treatment can induce the release of antimicrobial compounds in a broad range of fungi, which have active effects against Gram‐positive bacteria, such as *Staphylococcus (S.) aureus*.^73^ Another anticonvulsant, lamotrigine, and its derivatives, also showed antimicrobial activity predominantly against Gram‐positive strains such as *Bacillus subtilis* and *S. aureus* and mild activity against Gram‐negative bacteria.^74^ Given the limited remission rate of current available pharmacotherapy for BD, exploring the drug‐microbiome interactions seems to be a promising approach to promote drug development and innovation.

### 5.4 Microbiome‐targeted therapy

The concept of “Psychobiotics” was first proposed in 2013, referring to a class of probiotics inhabiting in the intestine that can benefit mentally ill patients.^5^, ^75^ Specific bacteria are capable of producing neuroactive substances such as SCFA, serotonin, and gamma‐aminobutyric acid, and possess antidepressant or anxiolytic activity along the MGB axis.^75^, ^76^ In euthymic patients with BD, 8‐week consecutive daily intake of probiotic supplement, a mixed combination of nine human bacterial strains belonging to genus *Lactobacillus* and *Bifidobacterium*, as an instruction recommended dose, improved GI quality of life, and decreased negative cognitive reactions.^77^ Moreover, the same research group further reported that 3‐month supplement with this probiotic formula could improve of performance concerning attention, psychomotor processing speed, and executive function in euthymic patients with BD.^78^ Another study suggested that adjunctive probiotic supplement (*Lactobacillus* GG strain and *Bifidobacterium lactis* strain) reduced the rehospitalization rate in patients who were recently discharged for mania.^79^ Although the exact mechanisms by which probiotic microorganisms benefit for mental health remain unknown, it is possible that they reconstruct the gut microbiota and modulate host immune reactions in response to various antigens.^75^ Although these studies are preliminary, they provided primary evidences that manipulating the gut microbiome, either supplement with probiotic bacteria or elimination of harmful bacteria, and thus have the potential as a therapeutic strategy in the prevention and/or treatment of BD. The primary task for developing microbiome‐targeted therapy is to identify specific gut bacteria or their metabolites linking to mental health in BD patients.

## 6 CONCLUSIONS

In conclusion, the growing evidence has indicated the close relationship between gut microbiome and mental health. Gut microbial alterations, manifesting as changed microbial diversity and communities, may be associated with disease characteristics of BD (*eg*, age of onset, different subtypes and phases) and treatment outcomes of pharmacotherapy. Tailoring to individual microbiome and clarifying its relation to the occurrence and development of BD help to uncover the pathogenesis of BD and facilitate early diagnosis. Microbiome‐targeted therapy aiming at promoting a harmonious host‐microbial symbiotic state seems promising to maintain mood homeostasis in BD patients. Great efforts are still awaited to figure out the gut microbial clues to BD.

## CONFLICT OF INTEREST

The authors declare no conflict of interest.

## AUTHOR CONTRIBUTION

All the authors contributed to the writing of this review. All the authors read and approved the final manuscript.
